# Design and Optimization of an MFL Coil Sensor Apparatus Based on Numerical Survey

**DOI:** 10.3390/s19224869

**Published:** 2019-11-08

**Authors:** Ali Azad, Namgyu Kim

**Affiliations:** Department of Civil and Environmental Engineering, Sejong University, Seoul 05006, Korea; g2016sfd008@sju.ac.kr

**Keywords:** magnetic flux leakage, coil sensor, numerical analysis, parametric study, non-destructive test

## Abstract

In this study, we aimed to design a coil sensor prototype capable of detecting metallic area loss based on numerical simulations using the magnetic flux leakage (MFL) method. Unlike previous numerical simulation-based studies, which are only conducted to obtain the MFL itself, the main objectives of this study were (1) to acquire the induced current in the coil sensor and (2) to optimize the apparatus based on a time-dependent numerical analysis. As a result, the optimum values of parameters in magnetizing and sensing units were obtained numerically. A magnetic sensor prototype was then fabricated using the optimum parameters obtained by numerical parametric study. Finally, experimental validation tests were conducted on a solid steel rod specimen with a stepwise cross-sectional reduction flaw. It was observed that numerical simulation had approximately 91% precision compared to the experimental test. The results reveal that application of a realistic numerical simulation of an MFL coil sensor can probably provide essential information for MFL-sensor fabrication and allows for preventive measures to be taken before manufacturing failure or defect misdetection.

## 1. Introduction

To assess the integrity of structures, structural health monitoring (SHM) for metal members, such as pipelines in the oil and gas industry, load carrying cables in bridges, lifts, cranes, etc., is an essential task and must be done regularly to eliminate any chance of structural failure and economic loss [1]. For instance, the current transport system for oil–gas products is a pipeline which requires routine inspections due to the presence of humidity and underground pressure, because any metal losses, cracks, and corrosions may cause an accident. Therefore, pipelines must always be functional while being completely sound and intact [2,3]. Steel cables are the main load-carrying elements in an enormous number of machines and structures. Although steel cables and ropes are designed to have high strength, cross-sectional damage and internal breakage and cracks can still occur during their service life and may compromise the entire structural integrity [4]. However, these types of damages are not easily detected by the naked eye and visual inspections (e.g., internal breakage of cable in cable-stayed bridges) [5]. Hence, demand for SHM using non-destructive testing (NDT) methods has increased significantly. Currently, there are a variety of conventional methods which are being applied based on non-destructive SHM principles such as visual inspection [6], magnetic flux leakage (MFL) [7,8], eddy current sensing [9,10], acoustic emission [11,12,13,14,15], and guided wave [16,17,18], among other techniques.

Traditionally, in the MFL method, the axial component of the magnetic flux density is used, which causes misdetection in the case of axial cracks. Liu et al. [19] proposed a circumferential excitation method to detect axial defects in pipelines. Also, they investigated the influence of a crack’s geometry (i.e., depth, width, and length) on the output signal. Li et al. [20] proposed a numerical simulation of the MFL inspection system for variety of inspection velocities and defect depths. In the 2D numerical simulation, the MFL was obtained by a reference line located right above the defect.

Jiang Xu et al. [21] investigated damage detection in artificially defected steel cables using both the MFL and magnetostrictive guided waves (MGWs) methods. They showed that the MFL method is capable of detecting internal damage (i.e., breakage and cross-sectional damage) for accessible parts of the specimen, while the MGW method can accurately detect unreachable defects such as breakage inside an anchor of a bridge cable. Johannes Atzlesberger et al. [22] tried to detect medium-size artificial defects in ferromagnetic specimens using MFL principles and a giant magnetoresistive (GMR) sensor. According to their experimental results, accurate detection, even in the case of small blemishes, was observed. Jianbo Wu et al. [23] mentioned that most of the conventional MFL sensors have an inevitable unwanted lift-off due to the non-ferromagnetic support layer between specimen and sensor. Consequently, by replacing this non-ferromagnetic support layer with a ferromagnetic one, they observed that the output signal of the detected leakage had a higher amplitude in comparison with conventional support. Runchuan Xia et al. [24] focused on corrosion detection using both experimental tests and a novel numerical formulation combined with a logistic curve in the case of self-magnetic flux leakage (SMFL) in the steel strand. Liu Xiucheng et al. [25] identified both surface and internal flaws in steel cable experimentally using a biased pulse magnetic field. In their approach, a tunnel magnetoresistive (TMR) sensor and a pair of coils sensors were implemented to detect surface and internal defects, respectively. Before conducting experiment, a conceptual numerical simulation was performed as well. In order to have an efficient magnetizing unit in a sensor probe, Wu et al. [26] optimized the magnetizing unit of an MFL sensor based on finite element analysis (FEA). In their 2D numerical simulation, the magnetic flux density was obtained using a cut-line which is representative of a TMR sensor. Further on, an experimental apparatus was made, and its performance was evaluated. However, in their numerical model, an actual sensor was not simulated and, more importantly, the parameters of the sensing unit were not optimized as well. To evaluate any changes in an MFL signal caused by the geometry of the magnetic circuit, Jaimes Saavedra et al. [1] performed a preliminary parametric simulation based on the MFL method. They concluded that, by increasing the thickness and reducing the length of the yoke, greater density flux leakage will be acquired. However, in their study, only a few parameters with a few corresponding values were considered and simulated, while other variables such as the strength of the generated magnetic field, sensor lift-off (i.e., the reference line’s lift-off in this study), etc., were disregarded. In order to address the lift-off issue caused by swinging in on-line monitoring of ferrous cables, Sun et al. [27] developed an open magnetic probe and compared it with a conventional magnetic yoke setup. To optimize the proposed magnetic apparatus, a simplified numerical simulation was conducted. However, in the numerical simulation, a sensing unit was not modeled and only the magnetic flux density was acquired using a reference line as the main goal of the numerical model.

The main objectives of previous studies related to damage detection using the MFL technique were to acquire the MFL signal only. To achieve this goal, a defected specimen was simulated in a simplified numerical model which, instead of defining the actual geometry of the sensor alongside its properties, a reference line as a representative of the sensor was defined in the air boundaries. As a result, a conventional numerical method can only obtain an MFL signal.

Moreover, possessing an optimized test setup is a necessary condition prior to conducting any experiment. Instead of performing experiments to obtain optimum values for critical parameters of a magnetic probe which is a substantially time-consuming process, numerical simulations are a good alternative. In previous numerical studies, due to the omission of the coil sensor in the numerical models, simplified parametric optimization was only focused on the magnetizer unit and supporting yokes.

Therefore, in this study, a parametric optimization using a precise numerical analysis was proposed. Instead of defining a reference line, actual geometry of the coil sensor along with its properties were defined. To acquire the induced voltage in the coil sensor corresponding to the MFL, a time-dependent analysis was performed. Furthermore, to provide an optimized magnetic test setup, it was decided to optimize the variables which were expected to have the most significant effects on the output signal. Thus, the optimum value of the variables, such as the required length of magnets, ferromagnetic handle’s length, magnitude of the magnetic flux density, coil sensor’s lift-off, permanent magnet’s lift-off, and number of wire loops in the coil sensor, were acquired based on the precise numerical simulation.

## 2. Principle of MFL Sensing for Metal Damage Detection

**Figure 1:**
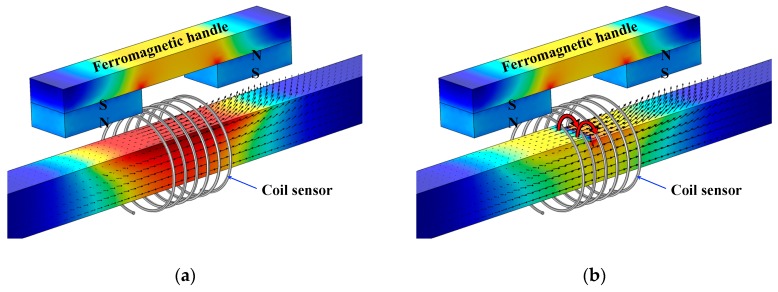
Magnetic flux leakage (MFL) phenomenon: (**a**) flawless specimen; (**b**) defected specimen.

One of the conventional NDT methods is the MFL method. This method is capable of detecting surface and internal damage such as breakage, corrosion, and cross-sectional damage [25]. The MFL technique includes a magnetizing unit, a ferromagnetic specimen, and a sensing unit to detect flux leakage from a specimen. Coil and hall-effect sensors are the sensing elements which generally identify cross-sectional loss and surface defect, respectively. When a defect-free specimen is being inspected with this method, due to the greater specimen’s relative permeability than air, magnetic flux will not leak from the surface of a specimen. On the other hand, when a damaged specimen is being inspected, magnetic flux will leak from a flaw (e.g., crack or discontinuity). Depending on the type of defect, this flux leakage can be detected by either coil or hall-effect sensors. Figure 1 illustrates the MFL phenomenon for both defect-free and stepwise cross-section loss specimens. Magnetic flux can be generated by either a permanent magnet or solenoid. Also, according to the principles of the MFL method, a specimen must be saturated [2,8,23]. To provide a strong magnetic field for magnetizing a specimen, both the number of coil turns and the amplitude of the current (either AC or DC) must be large enough. Despite the fact that the coil sensor can generate a uniform magnetic field, due to the high temperature and requirements of a strong power source, a permanent magnet is a good alternative, as it is a self-sustaining source of strong magnetic field. 

### Governing Equations

In order to conduct numerical analysis for one specific physic, relevant governing equations of that physic must be considered and satisfied. Thus, Maxwell’s equations with considering magneto-quasi-static approximation can be written as:

(1) $$\begin{array}{c} \nabla \times H=J,\\ \nabla \times E=-\frac{\partial B}{\partial t},\\ \nabla .B=0, \end{array}$$

where H, J, E, and B represent magnetic field intensity, current density, electric field, and magnetic flux density, respectively. According to Faraday’s law, the integral of the electric field over a closed loop is equal to the generated voltage inside the coil. Hence, the induced voltage inside a multi-turn coil is calculated as below [2,28]:

(2) $$\begin{array}{c} \oint E.ds=-N\frac{d\emptyset \left(t\right)}{dt},\\ V_{c}=-N\frac{d\emptyset \left(t\right)}{dt}, \end{array}$$

where *N* is the number of wire loops, ∅ is magnetic flux, and $V_{c}$ represents the induced voltage inside the multi-turn coil.

## 3. Optimization of an MFL Coil Sensor Apparatus Based on a Numerical Parametric Study

In order to fabricate a prototype coil sensor capable of detecting cross-sectional damage, a numerical study using the finite element model (FEM) should be performed. Therefore, the main aim of a numerical simulation is to obtain the optimum values of the essential parameters. By performing a numerical study rather than conducting trial and error, not only can additional costs be eliminated, but a significant amount of time and efforts can also be saved. In this study, FEA was performed using the COMSOL Multiphysics 5.3a commercial software by applying the AC/DC module to consider the effects of the magnetic field together with the deformed mesh physic and simulate specimen movement in a 2D axisymmetric geometry. It should be noted that, to consider current induction, Faraday’s law of induction which states the relationship between the magnetic field and electric field with respect to time, must be assumed as a necessary condition for numerical simulation. Thus, all simulations were time dependent with considering quasi-static approximation. 

**Figure 2:**
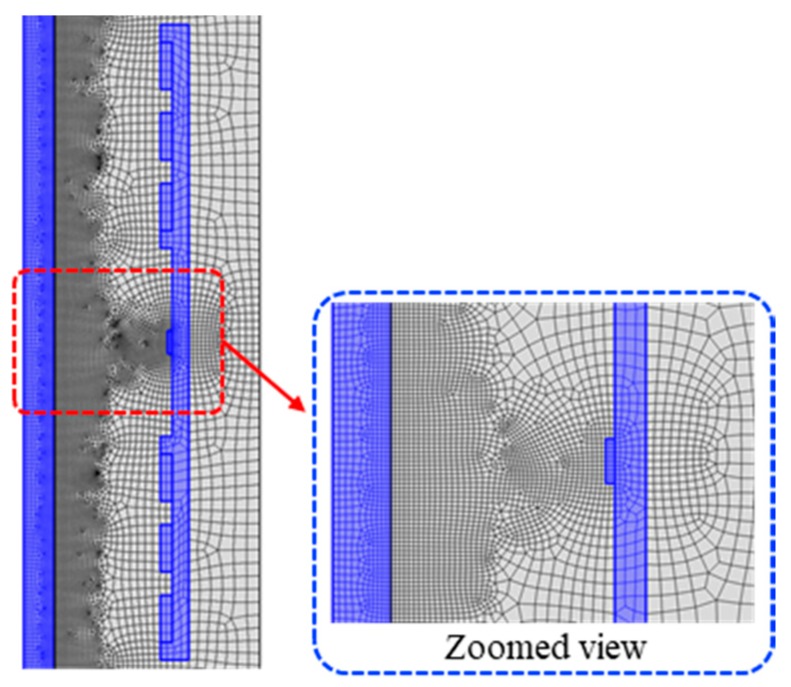
The meshed geometry (specimen, ferromagnetic handle, and permanent magnets are highlighted in blue).

To reduce time consumption and increase the accuracy of numerical model, mesh size and type must be adjusted based on the model. Meshed geometry of the model can be found in Figure 2. In this numerical model, a free quadrilateral mesh with a minimum element size of 1 mm and a maximum element growth rate equal to 1.2 were used to mesh the geometry.

**Figure 3:**
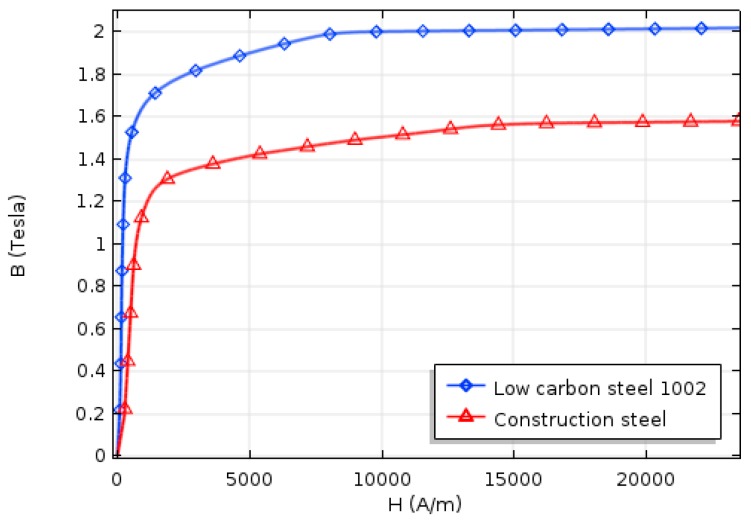
The B–H curves of a specimen (red line) and ferromagnetic handles (blue line).

In order to implement B–H curves of ferromagnetic materials, anhysteretic B–H curves of low carbon steel 1002 and construction steel, which are presented in the COMSOL library, were used as representatives of ferromagnetic handles and specimen, respectively. Plotted B–H curves of the specimen and handles can be found in Figure 3. It should be noted that, to reduce the complexity of the model in this study, remanence magnetization was disregarded. Moreover, by defining actual multi-turn coil sensor geometry in numerical simulation, instead of just obtaining flux leakage, the coil sensor’s signal was directly obtained in millivolt unit. For having better damage detection visualization, after obtaining the coil sensor’s output signal, it was proposed to perform an integration operation on the output signal. The integrated coil sensor’s signal allows for easier inspection independent of the specimen’s pace; also, each defect can have its own specific signature in the output signal.

**Figure 4:**
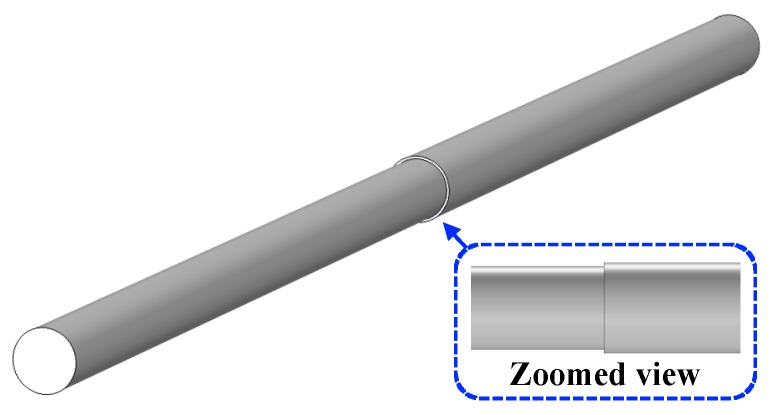
Specimen with a 15% cross-sectional loss (schematic view).

It is important to note that, to perform the parametric simulation, the simulated specimens in all cases had the same stepwise cross-sectional reduction with 15% cross-section loss which can be seen in Figure 4. Also, the specimens’ velocity was assumed constant and equal to 20 cm/s for all simulation models.

### 3.1. Design Parameters

Generally, an MFL sensor setup consists of two main parts: magnetizing and sensing units. To optimize the magnetic apparatus, both magnetizing and sensing units must be considered in the numerical model. In the magnetizing unit, any variation in the values of the parameters, such as length of the permanent magnets, magnitude of the magnetic flux density, length of the ferromagnetic handles, and lift-off of the permanent magnets, can significantly impact on the output signal. For instance, if the handle’s length is considered too long, not only does operating the apparatus become difficult due to the excessive weight and size, but the induced magnetic field inside the specimen in the vicinity of the sensor will also have less strength which eventually causes low signal amplitude. In addition, in the sensing unit, there are two important parameters that are expected to affect output signal substantially: the number of wire turns and lift-off from the surface of the specimen. For example, if the lift-off value is assumed too small, the signal amplitude increases; however, due to the small gap between the specimen and sensor, the chance of collision increases as well. Hence, the optimum values of important parameters in both sensing and magnetizing units must be evaluated. Table 1 shows the parameters that were considered in this parametric study.

### 3.2. Length of Permanent Magnets

Magnetic flux must be spread across region of interest in a specimen uniformly. Despite the fact that larger magnets induce a more uniformly distributed magnetic field, having too large permanent magnets will increase the weight of a sensor setup and occupy more space which makes it harder to operate the probe. Therefore, it was decided to identify the optimum length for the permanent magnets. In order to achieve this goal, seven different lengths with a constant distance to the center of the yoke were considered and simulated, while the magnitude of the magnetic flux density was assumed strong enough to saturate the specimen completely (according to the principal of the MFL method) [2,8,23] and all other parameters were considered to have constant values. These seven values were 2, 4, 6, 8, 10, 12, and 14 cm.

**Figure 5:**
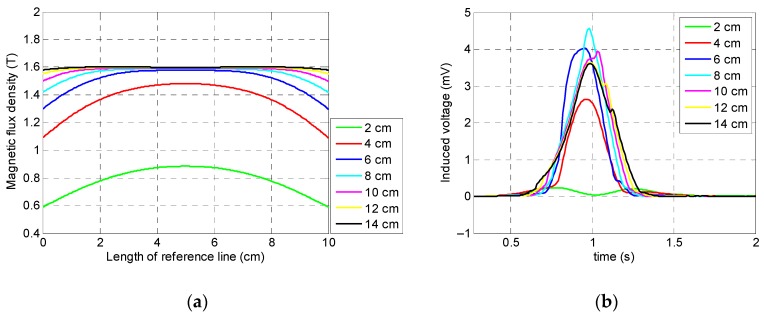
Numerical simulation results of the magnets’ lengths: (**a**) saturation status of the specimen; (**b**) coil sensor’s signal.

According to Figure 3, the specimen was saturated when the induced magnetization reached 1.3 T. Figure 5a illustrates the saturation status of the specimen for all seven lengths across the reference line which was located inside the specimen and the middle of the magnetic yokes. As expected, the specimen was saturated for almost all different lengths of the permanent magnets except for 2 cm which did not provide magnetic induction strong enough to saturate the specimen’s region of interest. Although longer magnets magnetized the specimen much more uniformly than shorter magnets, the longer lengths of the magnets will increase the weight, size, and final cost of the magnetic sensor. Also, shorter permanent magnets did not magnetize the specimen uniformly (Figure 5a). 

**Figure 6:**
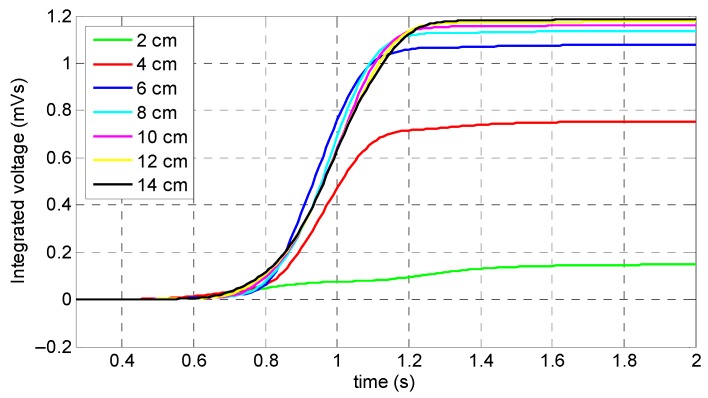
Integrated signals of the coil sensor corresponding to the magnets’ lengths.

The output signals of the coil sensor corresponding to the different lengths of the permanent magnets are presented in Figure 5b. It can be observed that all variables, except a 2 cm length, had a single peak behavior which is representative of MFL from a saturated specimen. Therefore, a 2 cm length could not be the optimum length due to the fact that the specimen’s saturation was not achieved. Despite the fact that the 4 cm length provided a magnetic field capable of saturating the specimen, it had the minimum measured value among all variables (except the 2 cm variable length) for both the direct coil sensor’s output signals and integrated signals which are shown in Figure 5b and Figure 6, respectively. As a result, the optimum length of the permanent magnets was 6 cm, because not only was the coil sensor’s peak signal one of the largest in amplitude, it was also the smallest, lightest, and, therefore, the most cost-friendly setup for damage detection. Thus, for convenient preparation of an experimental test setup, it was decided to use three magnets with a 2 cm length instead of a one-piece magnet with a 6 cm length.

### 3.3. Magnitude of the Magnetic Flux Density

Inspection using the MFL method requires a saturated specimen. Hence, this section aimed to evaluate the optimum value of the magnetic flux density of the permanent magnets. To achieve this goal, all other parameters, such as length of magnets (optimum value), magnets’ lift-off, etc., were modeled with constant values; the only variable was the magnitude of the magnetic flux density. If this value is chosen too small, the specimen is unlikely to be saturated and the chance of having detectible flux leakage will decreased dramatically. Also, if this value is chosen as too large, in spite of the fact that it would lead to a uniformly saturated specimen, significant drawbacks, such as a partially magnetized specimen and operation difficulty due to the excessive attraction force, will appear. 

Based on the optimum length of the magnets and the B–H curve of the specimen, nine values for the magnitude of the magnetic flux density were considered: 0.1, 0.25, 0.5, 0.75, 1, 1.25, 1.5, 3, and 5 T. Before starting the simulation, it was expected to have a saturated specimen around 1 T; as a result, the last couple of values were significantly larger than the initial values when comparing the effect of a fully saturated specimen with a specimen during the initiation of saturation.

**Figure 7:**
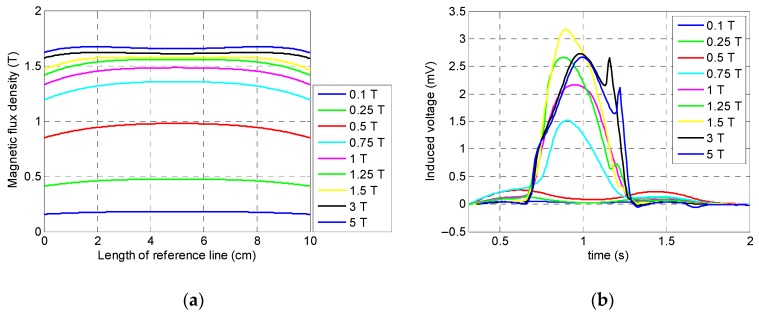
Numerical simulation results of the magnitude of the magnetic flux density generated by magnets: (**a**) saturation status of the specimen; (**b**) coil sensor’s signal.

**Figure 8:**
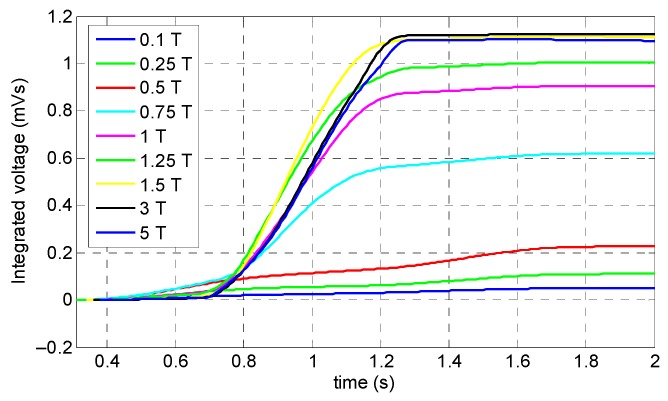
Integrated signals of the coil sensor corresponding to the magnitude of the magnetic flux density.

According to Figure 7, it can be seen that the specimen was not saturated when the magnitude of the magnetic flux density was less than 0.75 T, and, at this magnitude, the specimen was only saturated in the middle of the reference line and presented the smallest peak amplitude among the greater magnitudes of magnetic flux density. Consequently, in the case of small size defects, a misdetection may happen. Hence, 0.1, 0.25, 0.5, and 0.75 T cannot be the optimum value. On the other hand, above 1 T, the specimen was fully saturated, and the corresponding peak signal of the coil sensor was large enough. It can be observed that, based on Figure 7 and Figure 8, by increasing the magnitude of the magnetic flux density beyond 1.5 T, the integrated signals showed the same value without any increment in the amplitude of the signal. The reason behind this phenomenon is that by increasing the magnitude of the magnetic field beyond the saturation point, the induced magnetization remains almost constant. According to Figure 8, it can be observed that, when the magnitude of the magnetic flux density increased from 1 T to 1.5 T, the amplitude of the integrated signals increased slightly. Thus, for increasing the practicality and decreasing the attraction force of the magnets, the optimum value of the magnitude of the magnetic flux density was chosen as 1 T.

### 3.4. Length of Ferromagnetic Handles

In order to have an evenly distributed magnetic field across the specimen and, more importantly, to have an accurate output of data, this study aimed to evaluate the optimum distance among the arrays of the magnets in each yoke, or, in other words, to optimize the length of the ferromagnetic handles. If a handle’s length is chosen as too short, then it leads to a less uniform flux distribution that is followed by inaccurate results. Moreover, if this value is chosen as too large, it leads to a large sensor setup which is hard to carry and operate. For determining the optimum value of the handles’ length, eight different handle lengths were chosen as candidates: 21, 23, 25, 27, 29, 31, 33, and 35 cm. 

**Figure 9:**
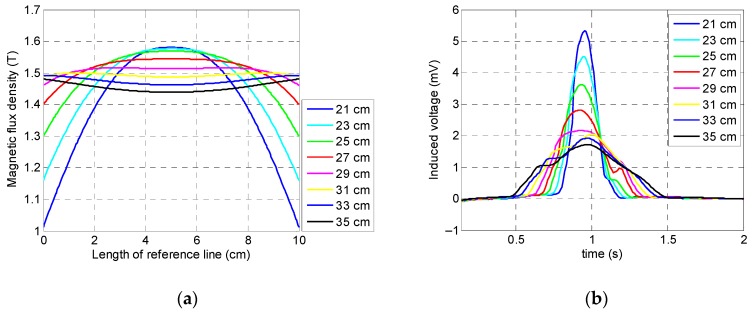
Numerical simulation results related to the length of the ferromagnetic handles: (**a**) saturation status of the specimen; (**b**) coil sensor’s signal.

As it can be observed in Figure 9a, the magnetization level was highly dependent on the handle’s length; a longer length of handle led to a much flatter curve of the induced magnetization in the specimen and, therefore, more reliable results due to the much more uniformly distributed magnetic field inside specimen. According to Figure 9b, the largest peak amplitude in the coil sensor’s signals belonged to the smallest handle (i.e., 21 cm) and the smallest peak amplitude belonged to the longest handle (i.e., 35 cm). Despite the shorter handles’ lengths (21, 23, and 25 cm) leading to greater signal amplitude, due to the considerable fluctuation in the induced magnetization within the reference line and less uniform saturation, these values cannot be considered as the optimum value. Although, in the cases of the 29, 31, 33, and 35 cm handle lengths a uniform magnetization across the reference line was obtained due to the small peak amplitude of the signal and, more importantly, handling and operating issues caused by the excessive size and weight, these values cannot be good representatives for the optimum value. Hence, the optimum value was chosen as 27 cm due to the fact that it had both a fairly strong peak amplitude and uniform saturation.

**Figure 10:**
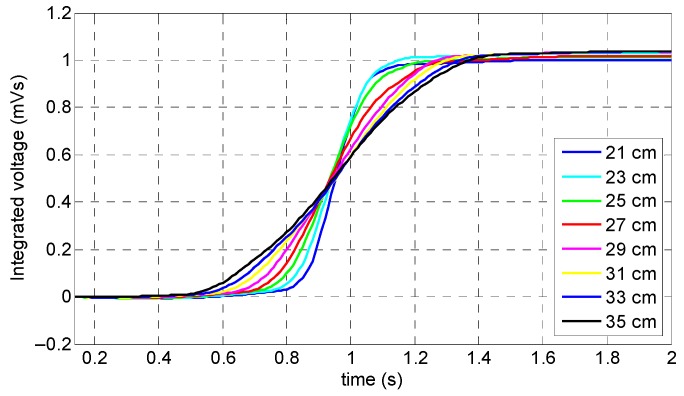
Integrated signals of the coil sensor corresponding to different lengths of the ferromagnetic handles.

As it can be observed in Figure 10, when the coil sensor’s signals were integrated, all eight signals presented the same peak amplitude (i.e., 1 mVs); the only differences that appeared were the different slopes corresponding to the different handles’ lengths—shorter handle lengths led to sharper slopes.

### 3.5. Lift-Off of Permanent Magnets

This section of study aimed to identify the optimum value of the permanent magnet’s lift-off which has a significant impact on output data. Lift-off of magnets can affect the saturation status of a specimen and can also change the amplitude of the output signal. For instance, if this value is chosen as too large, not only will the handling of the sensor setup be harder due to the larger size and additional weight of the sensor device, but the increasing distance between the magnets and the specimen will cause less magnetization to be induced in the specimen as well. On the other hand, making this gap too small leads to difficult inspection, since the specimen can stick to the magnet’s surface which might cause interruptions during inspection and sensor malfunctioning. Thus, optimizing a magnet’s lift-off is essential. Hence, it was decided to consider five different lift-off values: 1.5, 3, 4.5, 6, and 7.5 cm. 

**Figure 11:**
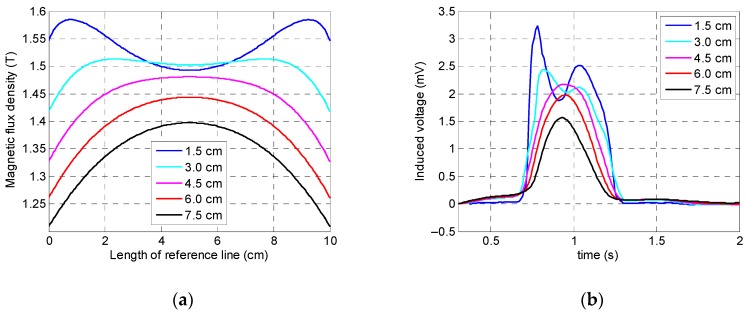
Numerical simulation results of the permanent magnet’s lift-off: (**a**) saturation status of the specimen; (**b**) coil sensor’s signal.

**Figure 12:**
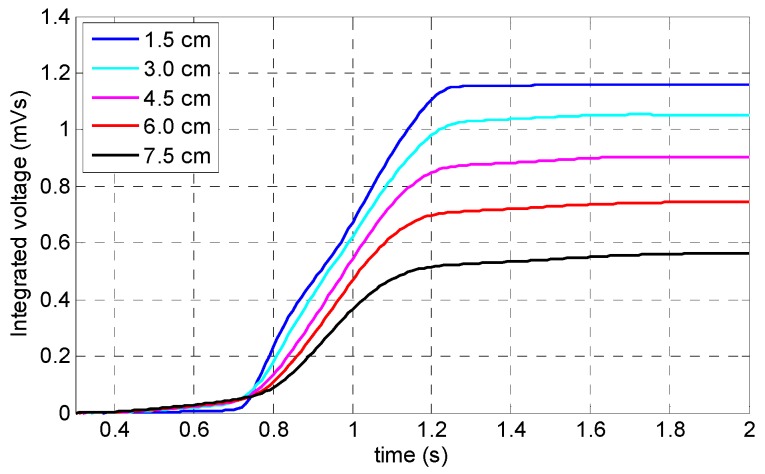
Integrated signals of the coil sensor related to the permanent magnet’s lift-off.

According to Figure 11, while the specimen was saturated for all lift-off values, there was a noticeable difference: induced magnetization was less uniformly distributed when the lift-off was greater than 4.5 cm. The coil sensor’s output signals obtained by simulation demonstrated that by decreasing the magnet’s lift-off value, the peak value of the signal increased as well. This behavior can be observed more clearly in Figure 12 which shows the integrated signals of the coil sensor. According to Figure 11b, a lift-off of both 1.5 and 3 cm both showed two local peaks instead of one peak behavior (which appears as detected damage using the MFL method); the reason behind this behavior is that when magnets are too close to the surface of the specimen, flux leakage will occur when the defect is located in the vicinity of the magnet’s arrays instead of the middle of the yoke (i.e., sensor position); therefore, two peaks will appear in the signal. To avoid this behavior, a lift-off value must be chosen greater than 3 cm. Moreover, having too large a lift-off value reduces the peak value considerably (e.g., 7.5 cm lift-off). As a result, 4.5 cm with a flatter magnetic induction curve was chosen as the optimum value of the permanent magnet’s lift-off.

### 3.6. Coil Sensor’s Lift-Off

In the previous sections, the length of the permanent magnets, magnitude of the magnetic flux density, length of the handle, and lift-off of the permanent magnets were obtained. Hence, in this section, the distance between coil sensor and the specimen, or, in other words, the coil sensor’s lift-off, was identified. It should be indicated that, if the coil sensor’s lift-off is too small, there is the possibility of unwanted collision between the specimen and the coil sensor which may cause irregularities in the output signal or, in severe cases, permanent sensor malfunctioning. On the other hand, if this value is too large, it can lead to inaccurate detection or even misdetection. To avoid these outcomes, a parametric study based on FEM is essential. Consequently, four different lift-off values were chosen based on the optimum value of the magnet’s lift-off: 1.2, 2.4, 3.6, and 4.8 cm. 

**Figure 13:**
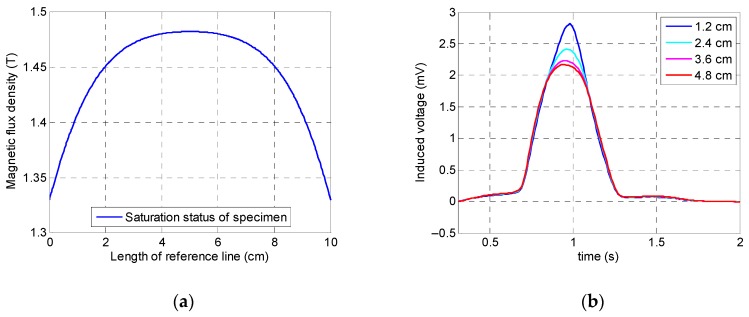
Numerical simulation results of the coil sensor’s lift-off: (**a**) saturation status of the specimen; (**b**) coil sensor’s signal.

**Figure 14:**
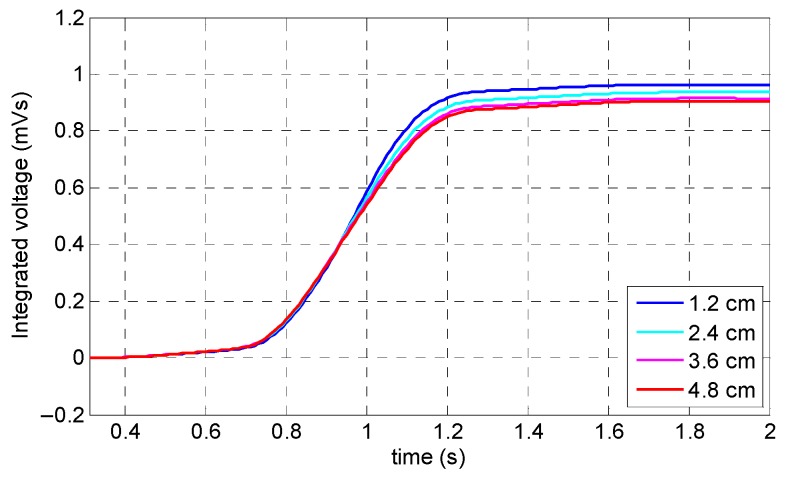
Integrated signals of the coil sensor corresponding to the lift-off of the coil sensor.

Figure 13a illustrates that the specimen was saturated with a relatively small fluctuation in the induced magnetization value. According to Figure 13b, as it could be expected, the largest peak value belonged to the minimum lift-off value (i.e., 1.2 cm). The rest of the variables had almost the same peak amplitude with slightly smaller values. This phenomenon can be observed better in the integrated signals of the coil sensor in Figure 14. Based on the fact that, by increasing the coil sensor’s lift-off up to the maximum value (which is located just below the yoke), not only did the output signal have an acceptable accuracy and precision, but by also implementing a coil sensor underneath the magnetic yoke, the chance of any collision between the specimen and sensor was eliminated. Consequently, the optimum value of the coil sensor’s lift-off was chosen as 4.8 cm.

### 3.7. Number of Coil Turns

Another important parameter in an MFL coil sensor is the number of the coil’s wire loops. Decreasing the amount of wire wound in a sensing coil not only leads to a much more compact coil with less occupied space, but also having a fewer number of turns reduces the thermal heat in a sensing coil. Usually, excessive thermal heat occurs when a DC current passes through an electromagnet due to the following reasons: too many turns; strong current running through the circuit; and long inspection times. It is necessary to mention that, often in damage detection using magnetic sensors (i.e., coil sensor), continuous inspection is required. Therefore, one way to reduce the aforementioned thermal heat is to decrease the number of wire loops in the coil sensor. 

Equation (2) shows the relationship between the induced voltage inside the coil sensor (V_c_), magnetic flux ∅(t), and the number of wire turns (N). As it can be observed, there is a linear relationship between the number of wire loops and the induced voltage. Therefore, adding to the number of wire loops will lead to a higher induced voltage, while reducing this number will cause a reduction in the induced voltage. Consequently, six different values were chosen as optimum value candidates: 1, 2, 5, 10, 15, and 20 loops.

**Figure 15:**
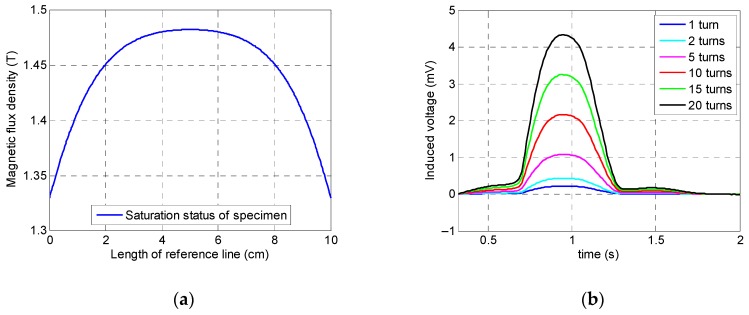
Numerical simulation results related to the number of turns in a coil sensor: (**a**) saturation status of the specimen; (**b**) coil sensor’s signals.

**Figure 16:**
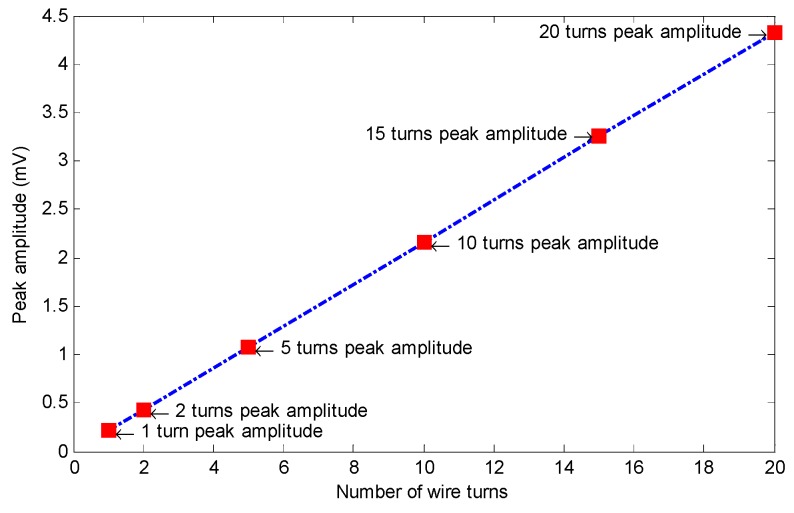
Linear relationship between the peak amplitude of the induced voltage and the number of wire turns.

**Figure 17:**
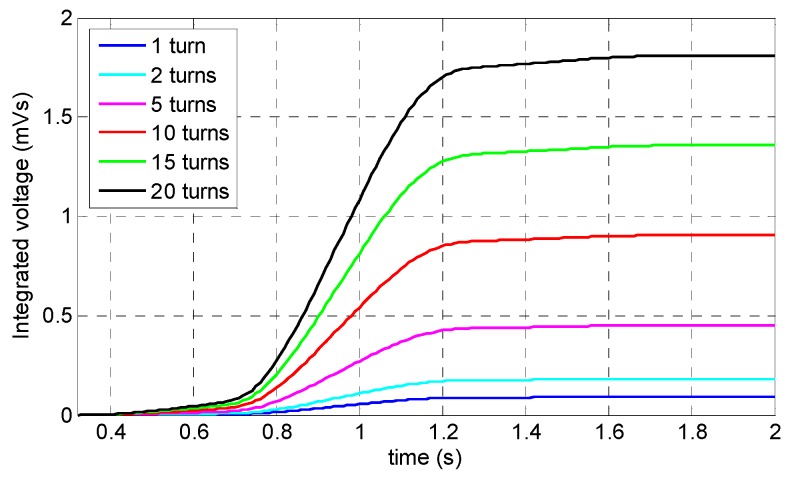
Integrated signals of the coil sensor corresponding to the number of the coil sensor’s turns.

Figure 15 shows the specimen’s saturation status along with the coil sensor’s signals corresponding to the different number of wire turns. Due to the magnetization level, the specimen was saturated with insignificant magnetization change. According to Equation (2) and Figure 16, which demonstrate the relationship between the peak amplitude of each signal and the number of wire loops, there is a linear relationship between the number of wire loops and the peak values of the coil signals. Thus, as it is illustrated in Figure 17, by increasing the number of loops in the sensing coil, the integrated voltage increased linearly with respect to the number of loops. Twenty wire loops provided the highest peak value while the single-turned coil sensor generated the minimum peak value. Despite the highest peak value belonging to the coil sensor with the 20 turns, excessive heating issues related to multi-turn coils with DC current may arise. In addition, by having a lower number of turns, it would be much more convenient to print the copper wires on a small- sized circuit board. Therefore, a coil sensor with 10 turns of wire can not only be easily printed and fit onto a small-sized circuit board, it can also identify small cross-sectional losses without difficulties. Thus, a coil sensor with 10 turns of wire was considered as the optimum value for the number of wire loops.

## 4. Prototype Fabrication of MFL-Sensing Apparatus Based on a Numerical Parametric Study

**Figure 18:**
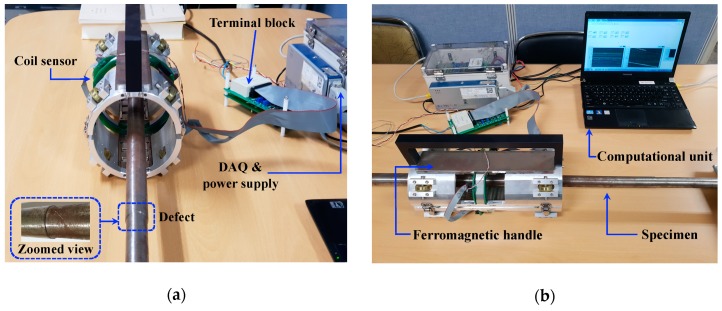
Magnetic apparatus, data acquisition (DAQ) system and specimen: (**a**) front view; (**b**) sideview.

**Figure 19:**
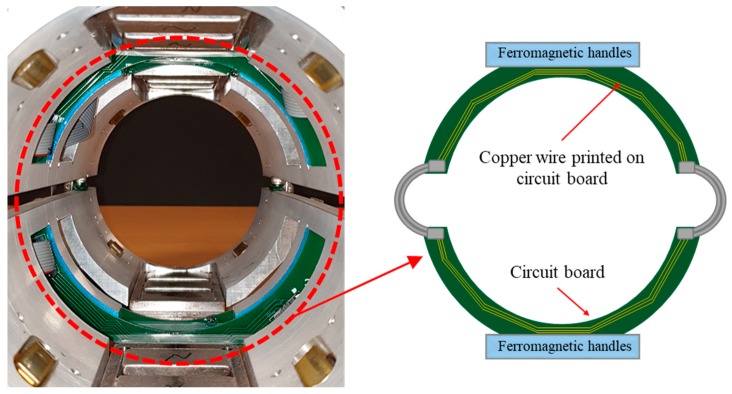
Schematic view of the coil sensor printed on a circuit board.

According to the optimum values obtained in previous sections as shown in Table 2, a prototype of an experimental sensor probe was fabricated as shown in Figure 18. This experimental test setup consisted of a pair of identical ferromagnetic handles made out of low carbon steel with optimum lengths equal to 27 cm, which were implemented symmetrically. Twelve permanent magnets were embedded in the ferromagnetic handles (based on the optimum length of the permanent magnet, three magnets were embedded into each side of the handles) and a sensing coil was printed on a silicon circuit board with a 4.8 cm lift-off and 10 loops of copper wire. To process the analog signal, a NI cDAQ-9181 data acquisitions system and a NI 9205 analog input module were used in the apparatus. Finally, the prototype magnetic probe was experimentally validated using an identical specimen which was equally modeled on the numerical simulations. Figure 18 and Figure 19 demonstrate the prototype magnetic probe along with a specimen made of construction steel with 15% stepwise cross-sectional reduction and a schematic view of the printed coil sensor on a circuit board, respectively.

**Figure 20:**
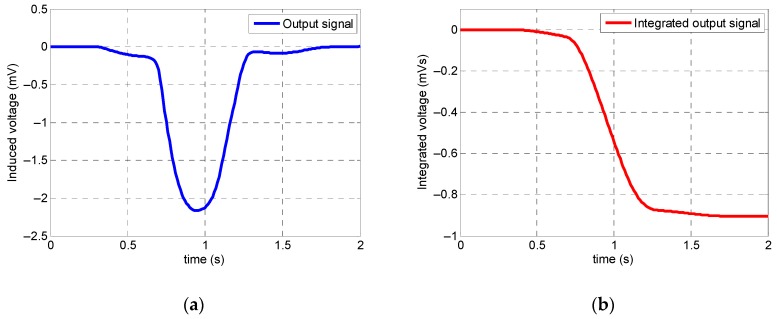
Optimum numerical simulation results of a steel specimen with 15% stepwise cross-sectional loss: (**a**) raw coil sensor signal; (**b**) integrated coil sensor signal.

**Figure 21:**
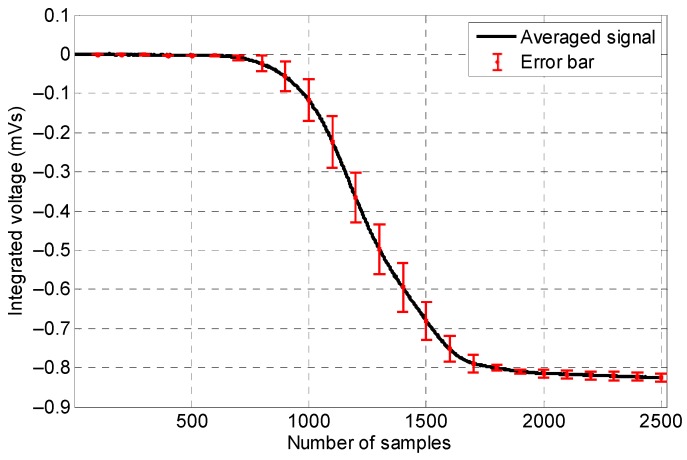
Experimental signal output obtained from the optimized experimental apparatus.

Figure 20 and Figure 21 illustrate the output signal of the coil sensor in the case of a specimen with 15% stepwise cross-section reduction obtained from the numerical simulation and experimental apparatus, respectively. As it was expected, there was only one peak corresponding to cross-section loss in the coil sensor’s output signal acquired from the numerical simulation. Both integrated signals from the numerical simulation and experimental test had the same distinctive defect pattern. Moreover, the integrated signal obtained from the coil sensor in the numerical simulation had approximately 91% precision with respect to the integrated signal acquired from the coil sensor in experimental test. Also, it can be noticed that the experimental apparatus was capable of detecting defects with high consistency.

## 5. Discussion and Conclusions

This study aimed to achieve an optimum design of a prototype coil sensor mounted on a magnetic probe which could be used to identify cross-sectional loss defects based on the MFL testing method. To do so, a parametric numerical study was performed, and the optimum values of the main parameters, such as length of permanent magnets, magnitude of the magnetic flux density, length of ferromagnetic handles, magnet’s lift-off, coil sensor’s lift-off, and the number of wire loops in the coil sensor, were obtained accordingly. The sensor probe prototype was made based on the optimum values acquired from the numerical simulations and, afterwards, the performance of the sensor probe was evaluated through an experimental test. 

In previous studies, due to the different units of the numerical model and experimental test output signals, conducting a comparative study with quantitative accuracy was impossible. In this study, both the numerical and experimental signals had the same physical units. As a result, accuracy of the numerical simulation was obtained as well. According to the high precision of the numerical simulation, which had a precision of approximately 91% with respect to the experimental test, not only was it confirmed that parametric optimization of a magnetic probe using numerical simulation can successfully optimize an experimental apparatus for cross-sectional defects, but it can also be used prior to any experimental tests in order to eliminate any chance of misdetection and inaccuracy due to the different defect types and sizes. 

In order to increase the accuracy of the numerical simulations, further study will be conducted by considering the global-search optimization algorithm and the exact material properties of a ferrite specimen and handles such as a dynamic B–H curve. 

## Figures and Tables

**Table 1:** Design parameters for parametric study.

Content	Magnetizing Unit				Sensing Unit	
Parameters	Length of Permanent Magnets	Magnitude of the Magnetic Flux Density	Length of Ferromagnetic Handles	Lift-Off of Permanent Magnets	Coil Sensor’s Lift-Off	Number of Coil Turns
Candidate Values	2 to 14	0.25 to 1.5	21 to 35	1.5 to 7.5	1.2 to 4.8	5 to 20
Intervals	2 cm	0.25 T	2 cm	1.5 cm	1.2 cm	5 turns
Additional Values	-	0.1, 3, 5 T	-	-	-	1, 2 turns

**Table 2:** Optimized design parameters.

Content	Magnetizing Unit				Sensing Unit	
Parameters	Length of Permanent Magnets	Magnitude of the Magnetic Flux Density	Length of Ferromagnetic Handles	Lift-Off of Permanent Magnets	Coil Sensor’s Lift-Off	Number of Coil Turns
Optimum Value	6 cm	1 T	27 cm	4.5 cm	4.8 cm	10 turns
